# Epidemiological status and risk factors of deep vein thrombosis in patients with femoral neck fracture

**DOI:** 10.1186/s13018-022-02926-8

**Published:** 2022-01-22

**Authors:** Chenhao Dou, Tianhua Li, Shuhong Yang, Qian Geng, Qing Lu, Yahui Zhang, Jingjing Yu, Fang Hu, Junqin Ding

**Affiliations:** 1 Department of Spine, Third Hospital of Hebei Medical University, Shijiazhuang, 050051 China; 2 Department of Nursing, Third Hospital of Hebei Medical University, Shijiazhuang, 050051 China; 3 The Second Operating Room, Third Hospital of Hebei Medical University, Shijiazhuang, 050051 China; 4 Department of Arthritis, Third Hospital of Hebei Medical University, Shijiazhuang, 050051 China; 5 Department of Orthopaedics, Third Hospital of Hebei Medical University, Shijiazhuang, 050051 China

**Keywords:** Deep vein thrombosis, Femoral neck fracture, Epidemiological status, Risk factors

## Abstract

**Objectives:**

The purpose of this study was to investigate the incidence of deep vein thrombosis (DVT) and clarify the risk factors of DVT in patients with femoral neck fracture.

**Methods:**

A self-designed questionnaire was used to collect the clinical data of 1209 patients with femoral neck fracture in our hospital from January 2019 to December 2019. The content of the questionnaire mainly includes general information, past medical history, history of present illness, operation related information, occurrence of DVT. The collected data were entered into Excel to analyze the incidence and risk factors of DVT in patients with femoral neck fracture. Chi square test and binary logistic regression model was used to screen the risk factors of DVT.

**Results:**

1209 cases of femoral neck fracture were included in this study. The incidence of DVT was 28.0% (339 patients). Among them, 71.7% (243 patients) were preoperative DVT and 28.3% (96 patients) were postoperative DVT. For the risk-factor analysis, gender, age, time from injury to hospitalization, operative method, anesthesia method and intraoperative blood loss were independent risk factors for DVT.

**Conclusion:**

The incidence of DVT in patients with femoral neck fracture is relatively high, and there are many related risk factors.

## Introduction

Femoral neck fracture is a common type of hip fracture. A cross-sectional study involving 27,462 people found that the incidence of femoral neck fracture in trauma patients is as high as 38.5% [1]. With the aging of population, the incidence of femoral neck fracture will further increase. Deep vein thrombosis (DVT) refers to the abnormal coagulation of blood in the deep vein and thrombus abscission can form pulmonary thromboembolism (PTE), which can lead to sudden death. It is reported that up to 300,000 people die of PTE every year in the United States [2]. As the most common complication of femoral neck fracture patients [3], DVT will not only increase the medical burden of society, but also threaten the lives of patients. Therefore, to be familiar with the epidemiological characteristics and risk factors of DVT and to prevent the occurrence of DVT are the focus of clinical work. Previously, a large number of studies have reported the incidence and risk factors of DVT in patients with hip fracture or lower extremity fracture [4–9]. However, due to the differences in demographic characteristics, preventive measures, diagnostic methods and other aspects, the reported results are quite different. In addition, there are few studies on DVT in patients with femoral neck fracture. In order to provide a theoretical basis for the prevention and management of DVT, this study investigated the patients with femoral neck fracture from two aspects of epidemiological characteristics and risk factors of DVT.

## Materials and methods

**General information** This retrospective study collected the clinical data of patients with femoral neck fracture who were hospitalized in our hospital from January 2019 to December 2019. A total of 1209 cases were included. Inclusion criteria were the following: (1) Age ≥ 14 years old; (2) After X-ray examination, it was diagnosed as femoral neck fracture. Exclusion criteria were the following: (1) Age < 14 years old; (2) Multiple fractures; (3) VTE was diagnosed before injury; (4) There was no deep vein ultrasound examination during hospitalization; (5) The length of stay was less than 3 days. In this study, patients without anticoagulant contraindications were given subcutaneous injection of low molecular weight heparin for prophylactic anti-coagulation; Patients without DVT were given antithrombotic pump (2 times a day, 30 min each time) for thrombosis prevention.

**Methods** The retrospective observational study was approved by the Ethics Committee of The Third Hospital of Hebei Medical University. According to the purpose of the study, combined with the literature and expert opinions, we designed the questionnaire. The content of questionnaire contained 6 aspects: general condition (gender, age, body mass index, etc.), history of past illness (diabetes, hypertension, respiratory system disease, cardiovascular and cerebrovascular disease, lower extremity vein disease, etc.), history of present illness, operation related information (operative mothod, duration of anesthesia, anesthesia mothod, etc.), disease diagnosis and the location of the thrombosis.

We retrieve the data contained in the questionnaire from the electronic medical record system. And enter the data into the EXCEL table for sorting and analysis. The epidemiological status and risk factors of DVT in patients with femoral neck fracture in 2019 were analyzed.

**Diagnostic methods and standards of DVT** Color Doppler ultrasound was used to diagnose DVT. The first time is within 24 h after admission and 3–5 days after operation. If the preoperative time is more than 7 days and the D-dimer is increased, an additional ultrasonic examination is needed before operation. If the postoperative bedridden time is more than 7 days, a weekly ultrasonic examination is needed. The non-surgical patients were examined within 24 h after admission and once a week. If the patient found lower extremity pain, swelling and other DVT symptoms, ultrasound examination should be performed at any time.

**Statistical methods** The Statistical Package for Social Sciences (SPSS) software version 21 (SPSS, Chicago, IL, USA) was used for statistical analysis. Measurement date are presented as means and standard deviations. Numeration data are presented as frequency counts and percentages. The χ^2^ test was usedin the comparison of numeration data. Binary logistic regression was used to analyze the independent risk factors. A *p *value of ≤ 0.05 was considered statistically significant.

## Results

### Epidemiology of DVT in patients with femoral neck fracture

1209 patients with femoral neck fracture were included in this study. The mean age and standard deviation was 65.9 ± 17.9 (range 14.0–96.0) years. The gender distribution was as follows: 433 males and 776 females. The incidence of DVT was 28%, among which the incidence of distal DVT was 25.3% and that of proximal DVT (popliteal and proximal vein thrombosis) was 2.7%. The incidence of intermuscular venous thrombosis was the highest (21.4%) (Table 1). Among them, 243 patients (account for 71.7%) were preoperative thrombus and 96 patients (account for 28.3%) were postoperative thrombus. 21 patients underwent inferior vena cava filter placement during hospitalization. No PTE occurred.

**Table 1:** Composition ratio and incidence of DVT in different parts of lower limbs

The location of the thrombosis	Number of patients (cases)	Composition ratio (%)	Incidence (%)
Thrombosis in intramuscular vein	259	76.4	21.4
Thrombosis in tibiofibular vein	47	13.9	3.9
Thrombosis in opliteal vein	18	5.3	1.5
Thrombosis in femoral vein	15	4.4	1.2
Thrombosis in iliac vein and above	0	0	0

### Univariate analysis of risk factors for DVT in patients with femoral neck fracture

Univariate analysis showed that gender, age, time from injury to hospitalization, hypertension, cardiovascular and cerebrovascular diseases, respiratory diseases, anesthesia duration, anesthesia method, operative method, intraoperative blood loss were associated with DVT (*P* < 0.05). However, there was no correlation between the cause of injury, fractured limbs, smoking history, diabetes, venous disease of lower extremity, tomour with DVT (*P* > 0.05) (Tables 2, 3).

**Table 2:** General information related to DVT in patients with femoral neck fracture

Variable	Thrombotic patients [cases (%)] 339 (28.0%)	Non thrombotic patients [cases (%)] 870 (72.0%)	*X*^*2*^	*P*
*Gender*			24.959	0.000
Male^a^	84 (19.4%)	349 (80.6%)		
Female^b^	255 (32.9%)	521 (67.1%)		
*Age (years)*			63.462	0.000
< 40^a^	3 (3.1%)	95 (96.9%)		
41–60^b^	28 (15.1%)	158 (84.9%)		
61–75^c^	148 (30.2%)	342 (69.8%)		
> 75^c^	160 (36.8%)	275 (63.2%)		
*Cause of injury*			1.572	0.456
Fall down	319 (28.0%)	820 (72.0%)		
Traffic injury	10 (23.3%)	33 (76.7%)		
High falling	10 (37.0%)	17 (63.0%)		
*Fractured limbs*			0.000	0.993
Left	177 (28.1%)	454 (71.9%)		
Right	162 (28.0%)	416 (72.0%)		
*Time from injury to hospitalization (days)*			15.479	0.000
< 1^a^	195 (24.5%)	602 (75.5%)		
1–6^a^	77 (33.3%)	154 (66.7%)		
≥ 7^b^	67 (37.0%)	114 (63.0%)		
*Smoking history*			0.037	0.848
Yes	17 (27.0%)	46 (73.0%)		
No	322 (28.1%)	824 (71.9%)		
*Diabetes*			0.808	0.369
Yes	70 (30.4%)	160 (69.6%)		
No	269 (27.5%)	710 (72.5%)		
*Hypertension*			6.820	0.009
Yes^a^	160 (32.1%)	339 (67.9%)		
No^b^	179 (25.2%)	531 (74.8%)		
*Cardiovascular and cerebrovascular diseases*			7.729	0.009
Yes^a^	159 (32.4%)	332 (67.6%)		
No^b^	180 (25.1%)	538 (74.9%)		
*Respiratory diseases*			7.043	0.008
Yes^a^	57 (37.0%)	97 (63.0%)		
No^b^	282 (26.7%)	773 (73.3%)		
*Venous disease of lower extremity*			1.441	0.230
Yes	3 (50.0%)	3 (50.0%)		
No	336 (27.9%)	867 (72.1%)		
*Tumour*			0.569	0.450
Yes	9 (34.6%)	17 (65.4%)		
No	330 (27.9%)	853 (72.1%)		

Use "a, b, c, ab" to indicate whether there is difference among groups, different letters represent the difference between groups with statistical significance, and ab represents the difference between group a and b with no statistical significance

**Table 3:** Surgical factors related to DVT in patients with femoral neck fracture

Variables	Thrombotic patients [cases (%)] 96 (11.2%)	Non thrombotic patients [cases (%)] 761 (88.8%)	*X*^*2*^	*P*
*Anesthesia duration (h)*			9.739	0.002
< 3^a^	35 (7.9%)	406 (92.1%)		
≥ 3^b^	61 (14.7%)	355 (85.3%)		
*Anesthesia method*				
General anesthesia^a^	52 (14.9%)	297 (85.1%)	14.746	0.001
Intraspinal anesthesia^a^	35 (11.7%)	297 (88.3%)		
Local anesthesia^b^	9 (4.3%)	199 (95.7%)		
*Operative method*				
Rreduction and internal fixation^a^	13 (3.4%)	368 (96.6%)	41.846	0.000
Joint replacement^b^	83 (17.4%)	393 (82.6%)		
*Intraoperative blood loss (ml)*			39.545	0.000
< 200^a^	17 (4.3%)	375 (95.7%)		
200–400^b^	51 (15.0%)	290 (85.0%)		
> 400^b^	28 (22.6%)	96 (77.4%)		

Use "a, b, c, ab" to indicate whether there is difference among groups, different letters represent the difference between groups with statistical significance, and ab represents the difference between group a and b with no statistical significance

The incidence of DVT in female patients was higher than that in male (*P* < 0.01). The incidence of DVT increased in patients older than 40 years and increased further in patients older than 60 years (*P* < 0.01). The incidence of DVT was significantly increased in patients with time from injury to hospitalization more than one week (*P* < 0.01). In the past history of patients, hypertension, cardiovascular and cerebro-vascular diseases, respiratory diseases and DVT were correlated. The incidence of DVT in patients with the above diseases was higher than that in patients without the above diseases (*P* < 0.01) (Table 2).

In this study, 857 cases of femoral neck fractures were treated with surgery. DVT occurred in 96 patients after surgery, the incidence was 11.2%. Among them, the incidence of DVT in patients undergoing joint replacement surgery was higher than reduction and internal fixation (*P* < 0.01). After anesthesia for more than 3 h, the incidence of DVT increased (*P* < 0.05). The incidence of DVT in patients undergoing general anesthesia and intraspinal anesthesia was higher than that in patients undergoing local anesthesia (*P* < 0.01). When the intraoperative blood loss was more than 200 ml, the incidence of DVT increased (*P* < 0.01) (Table 3).

### Logistic regression analysis of risk factors for DVT in patients with femoral neck fracture

The variables screened by Chi square test were analyzed by binary logistic regression. The variable assignment results was shown in Table 4. Five factors with statistical significance were included in logistic regression analysis. As shown in Table 5, logistic regression analysis showed that sex, age, time from injury to hospitalization,

**Table 4:** Variable assignment description

Variables	Variable name	Assignment description
X1	Gender	female = 1, male = 0
X2	Age	> 75 = 3, 61–75 = 2, 41–60 = 1, < 40 = 0
X3	Time from injury to hospitalization	≥ 7 = 2, 1- = 1, < 1 = 0
X4	Hypertension	Yes = 1, No = 0
X5	Cardio cerebrovascular diseases	Yes = 1, No = 0
X6	Respiratory diseases	Yes = 1, No = 0
X7	Operative method	Joint replacement = 1, Reduction and internal fixation = 0
X8	Anesthesia duration	≥ 3 = 1, < 3 = 0
X9	Anesthesia method	General anesthesia = 2, Intraspinal anesthesia = 1, Local anesthesia = 0
X10	Intraoperative blood loss	> 400 = 2, 200–400 = 1, < 200 = 0
Y	Whether the patient has DVT	Yes = 1, No = 0

**Table 5:** Logistic regression analysis of risk factors for DVT in patients with femoral neck fracture

Variable	B	S.E	Wald	*P*	Odd risk (OR)	95% CI	
						Minimum	Maximum
Gender	0.563	0.151	12.557	0.000	1.709	1.271	2.300
*Age (years)*			27.260	0.000			
41–60	1.527	0.624	5.977	0.014	4.602	1.354	15.648
61–75	2.184	0.605	14.416	0.000	8.886	2.717	29.056
> 75	2.444	0.607	16.191	0.000	11.522	3.503	37.896
*Time from injury to hospitalization (days)*			5.671	0.059			
1-	0.272	0.167	2.638	0.104	1.312	0.945	1.821
≥ 7	0.376	0.180	4.373	0.037	1.456	1.204	2.071
Operative method	1.370	0.329	17.308	0.000	3.963	2.064	7.504
*Anesthesia method*			8.299	0.016			
Intraspinal anesthesia	1.051	0.397	7.091	0.008	2.859	1.314	6.220
General anesthesia	1.069	0.383	7.792	0.005	2.913	1.375	6.170
*Intraoperative blood loss (ml)*			13.131	0.001			
200–400	0.888	0.316	7.909	0.005	2.431	1.309	4.516
> 400	1.358	0.378	12.917	0.000	3.887	1.854	8.151

operativ method, anesthesia method and intraoperative blood loss were independent risk factors of DVT in patients with femoral neck fracture.

### Comparison of hospitalization time and expenses of patients with femoral neck fracture

By comparing the hospitalization time and expenses of patients with femoral neck fracture, the results showed that: the hospitalization time of thrombotic patients (15.4 ± 11.2) was longer than that of patients without thrombus (11.5 ± 8.1) (*P* < 0.01); the hospitalization expenses of thrombotic patients (66,474.0 ± 34,533.3) were more than that of patients without thrombus (51,000.3 ± 32,088.0) (*P* < 0.01) (Table 6).

**Table 6:** Hospitalization time and expenses of patients with femoral neck fracture

	Thrombotic patients	Non thrombotic patients	*t*	*P*
Hospitalization time (day)	15.4 ± 11.2	11.5 ± 8.1	8.474	0.000
Hospitalization expenses (yuan)	66,474.0 ± 34,533.3	51,000.3 ± 32,088.0	8.199	0.000

## Discussion

Femoral neck fracture is a common type of traumatic fractures of the lower limbs, and venous thromboembolism, as a common complication of patients with femoral neck fractures, has attracted the attention of clinical medical workers and scientific researchers. Through retrospective research, this study analyzed and reported the incidence of venous thrombosis, the location of thrombosis, and common risk factors in patients with femoral neck fractures, so as to strengthen medical workers’ understanding of the epidemiological characteristics of venous thrombosis. And further do a good job in the prevention and management of venous thrombosis.

The incidence of DVT was 28.0% in patients with femoral neck fracture. The incidence of venous thrombosis at different sites was as follows: 21.4% of intermuscular vein, 3.9% of tibial and fibular vein, 1.5% of popliteal vein and 1.2% of femoral vein. Hu Wang et al. [4] reported that the incidence of proximal DVT in patients with femoral neck fracture was 6.5%. The incidence of proximal DVT in this study was 2.7%, significantly lower than that reported in the literature. It shows that in recent years, the majority of medical staff pay more attention to the management of venous thrombosis and the continuous improvement of the prevention measures of venous thrombosis, which has made remarkable achievements in reducing the incidence of venous thrombosis.

The incidence of intermuscular venous thrombosis was the highest (21.4%). Most scholars believe that intermuscular venous thrombosis is not enough to developinto life-threatening PTE [7, 10, 11]. In addition to anticoagulant therapy, there is no restriction on limb movement. Intermuscular venous thrombosis has not been included in the process of counting the incidence of venous thrombosis in many studies [3, 4]. Although the incidence of proximal vein thrombosis is lower than that of intermuscular vein thrombosis, the risk of proximal vein thrombosis is very high. Galanaud et al. [12] identified that the incidence of PTE related death in patients with proximal DVT within three months was 0.7% after standardized anticoagulant. Dahl OE [13] pointed out that the incidence of PTE related death after hip fracture operation is as high as 3.5%. So we should pay more attention to the proximal thrombus. Inferior vena cava filter is often placed in patients with proximal thrombosis before operation to effectively prevent the occurrence of PTE [14]. In this group, 33 cases of proximal thrombus were investigated, 21 cases were inserted into the filter before operation, and those without the filter were given anticoagulation, limb immobilization and other measures. Therefore, there was no PTE in our hospital in this investigation.

In the present study, gender, age, diabetes, hypertension, respiratory diseases, cardiovascular and cerebrovascular diseases, venous disease of lower extremity, operative method, anesthesia duration, anesthesia mothod, etc. were employed as risk factors multivariate analysis. Results showed that the independent risk factors of DVT were gender, age, time from injury to hospitalization, operative method, anesthesia method and intraoperative blood loss. In addition to the gender, the independent risk factors of DVT in this study were consistent with the risk items in Caprini score [3]. Combined with chronic diseases will also increase the risk of venous thrombosis in patients [15, 16]. Through analysis, this study found that hypertension, cardiovascular and cerebrovascular diseases, respiratory diseases are relating to DVT, but are not independent risk factors for DVT.

**Gender** The incidence of DVT in women is higher than that in men. In the past, it was reported that the incidence of DVT in men is higher than that in women, and the risk of DVT in men's life is 2.3 times higher than that in women [17, 18]. According to the analysis, the subjects included in this study are all patients with femoral neck fracture, while the elderly women are the high incidence population of femoral neck fracture. In this group, 663 women over 60 years old accounted for 85.4%, 276 men accounted for 62.3%, which may lead to the higher incidence of DVT in women with femoral neck fracture.

**Age** The incidence of DVT is increasing with the increase of age. When the age of patients is lower than 40, the incidence of DVT is lower. After 40, the risk of DVT will increase gradually. The incidence of DVT in patients over 60 is as high as 30.2%, which is consistent with the conclusion of most studies. Naess IA [19, 20] has reported that about 70% of DVT occurs in people aged 60 and over, and about 25% in patients aged 80 and over.

**Time from injury to hospitalization** The period of thrombus formation is relatively short, and the incidence of venous thrombus in passengers can be as high as 10% in a long flight over 8 h [21]. Through the analysis of the time form injury to hospitalization of the patients with femoral neck fracture, we found that 24.0% of the patients admitted within 24 h after the injury would be diagnosed with DVT, and 37.0% of the patients admitted for more than 7 days after the injury will have DVT, which may be related to that the patients do not pay attention to the prevention of thrombus in home or other basic hospital treatment. Therefore, DVT screening should be paid more attention to in patients with long time from admission to hospital after injury. Give early detection and treatment.

**Hypertension** Richad et al. [22] have reported that there is a significant positive correlation between blood pressure and whole blood viscosity in patients with hypertension, so the changes of blood viscosity and hypercoagulability in patients with hypertension is one of the reasons for the increased risk of DVT in patients with hypertension. In addition, blood pressure fluctuation may also cause vascular endothelial damage, exposure to collagen fibers, and increase the risk of DVT. In addition, blood pressure fluctuation may also cause vascular endothelial damage, exposure to collagen fibers, and increase the risk of DVT [23].

**Cardiovascular and cerebrovascular diseases** Cardiovascular and cerebrovascular diseases refer to the manifestations of general vascular diseases or systemic vascular diseases in the heart and brain. Like hypertension, it involves the changes of hemodynamics and hemorheology as well as the damage of blood vessel wall, so it will increase the risk of DVT to some extent. K ö nigsbr ü GGE o reports that patients with congestive heart failure are three times more likely to develop DVT than patients without congestive heart failure [24, 25].

**Respiratory diseases** A large number of studies have confirmed that patients with chronic obstructive pulmonary disease (COPD) have a higher risk of DVT. In addition to chronic obstructive pulmonary disease, the respiratory diseases included in this study include sleep apnea syndrome, chronic bronchitis, emphysema, etc. The results show that the above factors also increase the risk of DVT in patients. After analysis, it should be related to the patients with respiratory diseases often have limited activities, and some patients lie in bed for a long time, resulting in venous blood stasis, blood in a hypercoagulable state. In addition, the body is in a state of chronic hypoxia, which is easy to damage the vascular endothelial cells [26, 27]. In addition, patients with respiratory diseases will be accompanied by the change of negative pressure in the chest. When the negative pressure drops, the venous blood return of the lower extremities will be limited, which increases the risk of DVT.

**Diabetes** The impact of diabetes on DVT has been controversial. Some researches think that the damage of vascular endothelial cells in diabetic patients will accelerate the aggregation of coagulation factors, which will lead to the formation of thrombus, and diabetics have an increased risk of malunion, nonunion and reoperation [28]. To some extent, it will also increase the risk of DVT. However, some scholars found that the incidence of DVT did not increase in diabetics compared with non diabetics. Through the analysis of the risk factors of patients with femoral neck fracture, we found no correlation between diabetes mellitus and the formation of DVTHowever, diabetes is one of the more common chronic diseases in modern society. Therefore, the author thinks that we should also carry out large sample research or related basic experimental research on the relationship between diabetes and DVT, in order to verify the relationship between them and provide evidence for the management of clinical patients.

**Operative method** Reduction and internal fixation and joint replacement are the two most commonly used surgical methods for femoral neck fracture, among which joint replacement has a higher risk of DVT than the other two because of its long operation time and large trauma. The results of this study also showed that the operation mode was an independent risk factor for DVT.

**Anesthesia method** Anesthesia is also an independent risk factor for DVT in patients with femoral neck fractures, of which patients with general anesthesia and intraspinal anesthesia have a higher incidence of DVT. The operation that need general anesthesia and intraspinal anesthesia also needed for a long time. Soomro et al. found that when the anesthesia time was more than 45 min, the risk of DVT would be greatly increased [29]. In addition, it has been found that the platelet and fibrinogen content of patients undergoing general anesthesia will be significantly increased, and their prothrombin time is shorter, which is easier to cause DVT compared with intraspinal anesthesia [28, 29]. In this study, the incidence of DVT in general anesthesia patients was slightly higher than that in spinal anesthesia patients, but there was no statistical difference between them.

**Intraoperative bleed loss** Intraoperative blood loss was also an independent risk factor for DVT. After analysis, it should be related to the larger trauma, the longer exposure time and the increased release of inflammatory factors [30]. In the operation with a large amount of bleeding, hemostatic drugs will be used in the perioperative period, which willalso increase the risk of DVT to a certain extent. In addition, patients with excessive perioperative intraoperative blood loss will lead to blood concentration, increase the aggregation of red blood cells, change the shape of red blood cells, leading to blood flow disorders [31].

**Other** Caprini risk assessment scale lists both the history of venous diseases of lower extremities and tumor as the risk factors of venous thrombosis, and many scholars think that patients with history of venous diseases of lower extremities or tumor will increase the risk of venous thrombosis. Although the results of the study showed that the incidence of DVT in patients with the above diseases is high, but because the sample size of patients with the above diseases in this study is small, there is no statistical difference, which needs further study of large sample size.

In addition, by analyzing the length of hospitalization and cost in two groups of people, we found that compared with non DVT patients, the length of hospitalization in DVT patients will be longer, and the cost of DVT patients will also increase. In addition, DVT patients need to extend the use of anticoagulants after discharge. Most of the proximal DVT patients need to put in the inferior vena cava filter before operation and need to be hospitalized again to take out the filter, all of which need to increase the additional cost. Therefore, from the perspective of economy and hospital bed turnover, the prevention and control of DVT should be done well in the early stage of admission to reduce the incidence of DVT. It is beneficial to reduce the economic burden of patients and the medical burden of the whole society.

There are limitations in this study. The follow-up data of discharged patients were not collected; the statistics of patients' intraoperative complications and other related information were not perfect; the sample size was small, and the preoperative and postoperative data were not discussed and analyzed in a stratified manner.

## Conclusion

The incidence of DVT in patients with femoral neck fracture is high and there are many risk factors. The independent risk factors of DVT in this study include: gender, age, time from injury to hospitalization, operative method, anesthesia method, intraoperative blood loss. In order to improve the detection rate of DVT and reduce the incidence of DVT, it is very important to identify the risk factors of DVT formation in patients with femoral neck fracture, combined with related laboratory and imaging examination. This study is a single center study, only included the patients with femoral neck fracture in our hospital in 2019 and the results have certain limitations. In addition, there are many factors included in this study. Due to the limitation of sample size, no effective stratified analysis can be carried out, and more comprehensive data still need to be studied in large sample and multi-center study.

In addition, due to the electronic system of our hospital, this study also failed to analyze the impact of specific surgical methods on venous thrombosis, which is also the deficiency of this study and needs to be paid attention to in the follow-up.

## Data Availability

The datasets generated and analyzed during the current study are available from the corresponding author on reasonable request.

## Abbreviations

COPD: Chronic obstructive pulmonary disease
DVT: Deep venous thrombosis
PTE: Pulmonary thromboembolism
